# Smoking prevalence and economic crisis in Brazil

**DOI:** 10.11606/s1518-8787.2021055002768

**Published:** 2021-03-29

**Authors:** Luis Eugenio de Souza, Davide Rasella, Rafael Barros, Erick Lisboa, Déborah Malta, Martin Mckee

**Affiliations:** I Universidade Federal da Bahia Instituto de Saúde Coletiva SalvadorBA Brasil Universidade Federal da Bahia. Instituto de Saúde Coletiva. Salvador, BA, Brasil; II Universidade Federal da Bahia Escola de Enfermagem SalvadorBA Brasil Universidade Federal da Bahia. Escola de Enfermagem. Salvador, BA, Brasil; III Universidade Federal da Bahia Programa de Pós-Graduação em Saúde Coletiva SalvadorBA Brasil Universidade Federal da Bahia. Programa de Pós-Graduação em Saúde Coletiva. Salvador, BA, Brasil; IV Universidade Federal de Minas Gerais Escola de Enfermagem Belo HorizonteMG Brasil Universidade Federal de Minas Gerais. Escola de Enfermagem. Belo Horizonte, MG, Brasil; V London School of Hygiene and Tropical Medicine London UK London School of Hygiene and Tropical Medicine. London, UK

**Keywords:** Tobacco Use Disorder, epidemiology, Poverty, Health Impact Assessment, Financial Management

## Abstract

**OBJECTIVE:**

To estimate the impact of the 2015–2018 economic crisis on tobacco consumption in Brazil.

**METHODS:**

This is an interrupted time series analysis conducted with data from 27 cities collected by VIGITEL, using linear regression models to account for first-order autocorrelation. Analyses were conducted based on gender, age group, and education level.

**RESULTS:**

Smoking rates decreased between 2006 and 2018, decelerating after the crisis onset. Differently than women, men showed an immediate but transient increase in smoking, followed by a decelerated decrease. Those over 65 also showed increased smoking rates immediately after the economic crisis onset, but decline accelerated later on. In turn, we found a trend reversal among those aged 31–44. Rates also decreased among those with lower education levels, but decelerated among those with more years of schooling.

**CONCLUSION:**

An economic crisis have varied impacts on the smoking habits of different population groups. Tobacco control policies should entail a detailed understanding of smoking epidemiology, especially during an economic crisis.

## INTRODUCTION

Worldwide, about 1.1 billion people are estimated to smoke, within which four out of five live in low- and middle-income countries. In 2015, smoking was the second leading risk factor for premature death and disability, accounting for more than 5 million deaths every year since 1990^1^.

In 2018, 9.3% of the 157.2 million Brazilians aged 18 and above were estimated to smoke (12.1% males and 6.2% females). In the same year, 2.4% of adults were considered heavy smokers (20 or more cigarettes/day), while 7.6% were exposed to passive smoking at home and 6.8% at work^2^.

In 2014, Brazil saw the loom of an economic crisis that caused the gross domestic product (GDP) to decline by over 8%^3^and unemployment to rise to 11.8% of the economically active population in the second quarter of 2019^4^. Consequently, governments have imposed severe austerity measures since 2015, applying deep cuts in social welfare programs. Despite major consequences for the vulnerable population^5^, such measures weakened the government’s regulatory capacity, including tobacco control^6^.

Smoking rates are commonly affected by a country economic situation in many different ways. Individuals experiencing anxiety may adopt behaviors that provide short-term relief, such as drinking alcohol^7^ or smoking^8^ – a mechanism termed tension reduction^9^. Conversely, cigarettes purchasing behaviors are elastic and rely on income^10^, so that consumption is expected to decrease alongside reduced disposable income, as cigarettes become relatively less affordable. As illustrated by the post-2008 financial crisis, the overall effect of crisis on smoking behaviors can be difficult to predict. In Greece, consumption decreased with reduced income and rising prices^11^. In the U.S., a study found smoking rates to decrease among employed individuals but to increase among those unemployed^12^, whereas a different research reported changes to vary according to age group^13^. A study conducted in Italy found an overall increase in smoking during the crisis^14^. Yet, all these studies were conducted in high-income countries.

Our study aims to determine whether smoking prevalence trends have been affected by the 2014 economic crisis among the overall and in different groups of the Brazilian population, a middle-income country.

## METHODS

### Study Design

This is an ecological time-series study conducted with the overall population of the 26 state capitals and the Federal District of Brazil, according to age, gender, and education level, during the period of 2006 to 2018.

Data were collected and published by the Brazilian Ministry of Health using the telephone-based surveillance system for risk and protective factors for chronic diseases (VIGITEL), which monitors the frequency and distribution of chronic noncommunicable diseases (NCDs) and associated risk and protective factors in 27 cities. Monitored conditions include diabetes, cancer, and cardiovascular and respiratory diseases, whereas risk and protective factors include smoking, alcohol and food consumption, obesity, and physical activity, as well as cancer screening data^2^.

From its implementation, in 2006, until 2018, VIGITEL conducted thirteen annual surveys, each with an average of 54,000 people. Sampling and data collection procedures aim to obtain probabilistic samples of the adult population (≥ 18 years old) residing in households served with fixed lines. VIGITEL also uses post-stratification weights based on gender, age, and education level, enabling comparison with each capital population distribution. Its minimum sample size is approximately two thousand individuals for city.

Smoking rate is estimated by the number of smokers divided by the number of interviewees. We deemed as smoker any individual who answered the question “Do you currently smoke?” positively, regardless of the number of cigarettes, frequency, and smoking duration.

The outcome analyzed was yearly smoking prevalence, and the denominator was the 27 capital cities estimated population. We compared each year smoking prevalence for two periods: 2006–2014 (pre-crisis) and 2015–2018 (during-crisis).

Data on smoking prevalence were stratified by gender (men and women), age group (18–30, 31–44, 45–64, and ≥ 65 years), and years of education (0 to 8 years, 9 to 11 years, and 12 or more years).

### Statistical Analysis

This is an interrupted time series (ITS) analysis conducted with Prais-Winsten linear regression models and robust standard errors. ITS is amongst the most robust approaches for measuring the effects of sudden political/economic or natural events when time-series data are available. It estimates two main coefficients: the immediate change in prevalence due to rapid changes (in our case, the rapid decrease in gross domestic product - GDP and increase in unemployment in 2015), and differences between slopes before and after the crisis, depicting long-term and gradual changes in prevalence^15^. Prais-Winsten linear regression models account for first-order autocorrelation of observations, which was verified for each model using Durbin-Watson statistics^16^. The time-series analysis comprehends the period from 2006 to 2018. The first segment of data, from 2006 to 2014, covers a period characterized by economic growth (in 2009, GDP showed a slight decrease of 0,2%), whereas the second segment comprehends the period up from 2015, when the country saw a looming economic crisis that would last until 2018, according to GDP decrease or stagnation or unemployment increase.

Although the economic recession in Brazil have technically started in mid-2014^17^, the imbalance in the economy and the consequent economic crises only became evident in 2015. In this scenario, the country was suitable for an ITS analysis. The country’s GDP continued to increase (0.5%) in 2014, but decreased by 3.5% in 2015 and further 3.3% in 2016. That was the second most severe recession in Brazil’s history, followed by the slowest recovery ever^18^. In the first quarter of 2017, GDP showed the first increase (by 1%) after eight consecutive quarterly decreases. However, the economy retreated again in the first quarter of 2019 after a period of weak growth, bringing the country’s GDP back to the level recorded in 2012^19^.

Unemployment rates increased from 6.8% in 2014 to 8.5% in 2015 and 11.5% in 2016, reaching its peak in 2017, when 14.2 million people were unemployed (13.7% of the workforce). Young people were the most affected: while the overall unemployment rate was 12.7% in the first quarter of 2019, it was 27.3% among people aged 18–24 years^4^.

Sensitivity analyses were performed to test different model specifications and assumptions, including Newey-West models^15^, showing similar results to those reported below.

## RESULTS

We found a sharp decrease (41.8%) in smoking prevalence from 2006 to 2018 among the adult population of Brazil’s 26 state capitals and the Federal District, from 16.2% in 2006 to 9.3% in 2018. Regarding gender, men presented a 40.4% reduction, from 20.3% to 12.1%, whereas women showed a 46% reduction, from 12.8% to 6.9%^20,2^.

As shown in Table 1, the average annual variation in smoking prevalence for the overall population was -0.63 percentage points [95%CI -0.76, -0.51] in 2006–2014, a period of economic growth. This decline was greater for men (-0.79 percentage points [95%CI -0.94, -0.64]) than for women (-0.49 percentage points [-0.68, -0.31]).

**Table 1:** Interrupted time series analysis of yearly smoking prevalence during the 2006–2018 economic crisis (recession) among Brazilians aged 18 years and older residing in one of 27 capital cities, according to gender.

	Overall population	Men	Women
Yearly average percentage variation of smoking prevalence in the 2006–2014 period	-0.63^a^	-0.79^a^	-0.49^a^
	[-0.76 to -0.51]	[-0.94 to -0.64]	[-0.68 to -0.31]
Yearly average percentage variation of smoking prevalence in the 2015–2018 period	-0.36^a^	-0.12	-0.45^a^
	[-0.62 to -0.10]	[-0.44 to 0.18]	[-0.60 to -0.29]
Level change in 2015 *vs* predicted level without economic crisis	0.19	0.13	-0.03
	[-0.39 to 0.77]	[-0.92 to 1.19]	[-1.03 to 0.95]
Difference in trends (2015–2018 period – 2006-2014 period)	0.27	0.66^a^	0.04
	[-0.02 to 0.58]	[0.33 to 1.00]	[-0.26 to 0.34]

95% confidence intervals in brackets.

^a^ p < 0.05.

Models considered autocorrelation and used robust standard errors to account for repeated measures.

During the economic crisis (2015–2018), the average annual variation in smoking prevalence was still negative (-0.36 percentage points [95%CI -0.62, -0.10]), but decelerated in comparison to the previous period for the overall population, men (-0.12 percentage points [95% CI -0.44, 0.18]) and also women (-0.45 percentage points [-0.60, -0.29]).

Considering data for this second period based on gender, men showed an immediate increase in smoking prevalence in 2015, suggesting that smoking prevalence would be 0.13 percentage points lower if the average annual decline of the 2006-2014 period had been maintained in 2015. Such immediate increase was accompanied by a marked decelerated decrease, so that the 2015–2018 yearly average percentage variation showed a 0.66 percentage point-difference in relation to that of 2006–2014.

Conversely, women presented a small decrease (-0.03 percentage points) in smoking prevalence in 2015, immediately after the crisis onset, compared to that of the previous trend. As with men, the second period showed a decelerated decrease (0.004 percentage points), although not statistically significant.

All age groups showed a decrease in smoking rates, although with varied patterns. Among those aged 18–30, rates decreased from 12.9% in 2006 to 7.6% in 2018; for those aged 31–44, the decline was from 17.2% to 9.7%; and for those aged 45–64 it was from 19.7% to 11.6%. The relative decline was between 41% and 43% in each case, but slightly lower in the oldest group, which reached a value of 35.7% (from 9.5% at baseline to 6.1%).

**Figure 1:**
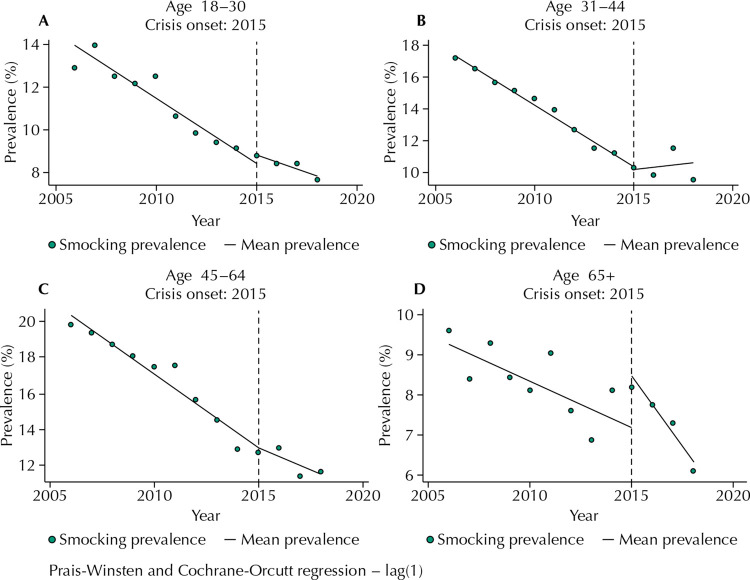
Annual prevalence of smoking in Brazilians aged 18 and older years residing in one of 27 capital cities between 2006 and 2018, by age-group.

As shown in Table 2 and Figure 1, the oldest group (65 years or older) showed a significant but transient increase in smoking rates immediately after the crisis onset. If the 2006–2014 average annual decline had been maintained in 2015, smoking prevalence would be 1.25 percentage points lower than that. Over the second period (2015–2018), decrease accelerated among this group, showing an average annual variation of -0.70 percentage points [-0.84, -056] in relation to that recorded for 2006–2014, of -0.22 percentage points [-0.34, -0.11].

**Table 2:** Interrupted time series analysis of yearly smoking prevalence during the 2006–2018 economic crisis (recession) among Brazilians aged 18 years and older residing in one of 27 capital cities, according to age group.

	18–30 years old	31–44 years old	45–64 years old	65 and older
Yearly average percentage variation of smoking prevalence in the 2006–2014 period	-0.61^a^	-0.75^a^	-0.81^a^	-0.22^a^
	[-0.75 to -0.48]	[-0.82 to -0.69]	[-1.05 to -0.58]	[-0.34 to -0.11]
Yearly average percentage variation of smoking prevalence in the 2015–2018 period	-0.34^a^	0.07	-0.50^a^	-0.70^a^
	[-0.45 to -0.23]	[-0.31 to 0.46]	[-0.87 to -0.14]	[-0.84 to -0.56]
Level change in 2015 *vs* predicted level without economic crisis	0.43	-0.12	0.03	1.25^a^
	[-0.28 to 1.16]	[-.06 to 0.81]	[-.36 to 1.43]	[0.30 to 2.20]
Difference in trends (period 2015-2018; period 2006–2014)	0.27^a^	0.83^a^	0.30	-0.47^a^
	[0.12 to 0.42]	[0.45 to 1.21]	[-0.16 to 0.78]	[-0.61 to -0.34]

95% confidence intervals in brackets.

^a^ p < 0.05.

Models considered autocorrelation and used robust standard errors to account for repeated measures.

Individuals aged 18–30 and 45–64 also showed an increase in smoking rates in 2015, unlike those aged 31–44, who presented lower rates than those predict if the previous trend was maintained. These changes were not statistically significant.

By comparing smoking rates variation before and after the economic crisis onset, we verified that the decrease in smoking rates decelerated within the youngest group (annual average of -0.61 percentage points before x -0.34 after) and in the 45-64 year-old group (-0.81 x -0.50). However, those aged 31-44 (-0.75 x 0.07) presented a trend reversal, with increasing prevalence.

Smoking rates decreased in all education levels, albeit from quite different starting points. Those with 0–8 years of education showed a decrease from 19.1% in 2006 to 13% in 2018, a 31.9% relative decrease; among those with 9–11 years of education, it decreased from 13.7% to 8.8%, a 35.7% decrease; and among those with 12 and more years, it decreased from 11.6% to 6.2%, equal to a 46.5% decrease.

**Figure 2:**
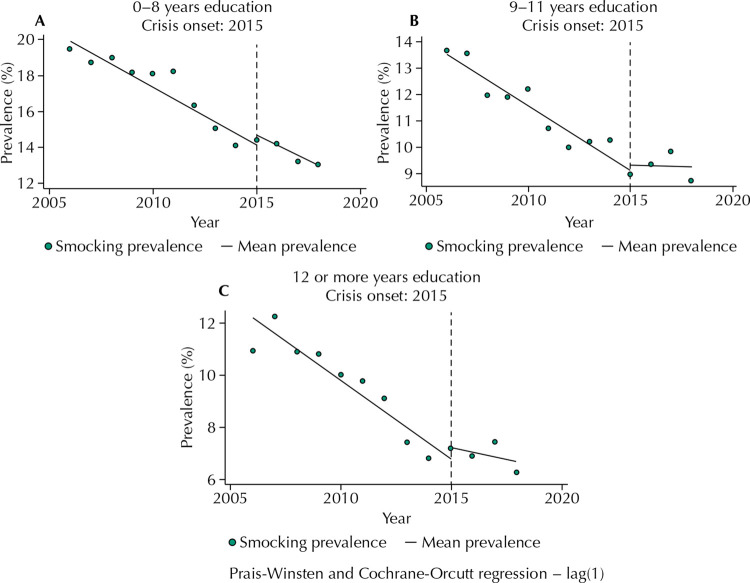
Annual prevalence of smoking in Brazilians aged 18 or older residing in one of 27 capital cities between 2006 and 2018, by education level.

The overall picture becomes more complex when comparing the two periods (Table 3 and Figure 2). All three groups present a decelerated decrease in smoking prevalence during the economic crisis when compared to the earlier period of economic growth, although not statistically significant in the least-educated group. We found the annual average variation of smoking prevalence to decrease from -0.64 to -0.55 in the group with 0–8 years of education, from -0.48 to -0.02 in the group with 9–11 years of education, and from -0.59 to -0.18 in the group with 12 years of education or more.

**Table 3:** Interrupted time series analysis of yearly smoking prevalence during the 2006–2018 economic crisis (recession) among Brazilians aged 18 years and older residing in one of 27 capital cities, by educational level.

	0–8 years of education	9–11 years of education	12 years and more of education
Yearly average percentage variation of smoking prevalence in the 2006–2014 period	-0.64^a^ [-0.88 to -0.39]	-0.48^a^ [-0.63 to -0.34]	-0.59^a^ [-0.83 to -0.36]
Yearly average percentage variation of smoking prevalence in the 2015–2018 period	-0.55^a^ [-0.89 to -0.21]	-0.02 [-0.33 to 0.27]	-0.18 [-0.46 to 0.09]
Level change in 2015 *vs* predicted level without economic crisis	0.56 [-0.48 to 1.61]	0.19 [-0.84 to 1.23]	0.42 [-0.90 to 1.76]
Difference in trends (2015–2018 period; 2006–2014 period)	0.08 [-0.42 to 0.59]	0.45^a^ [0.14 to 0.77]	0.41^a^ [0.07 to 0.75]

95% confidence intervals in brackets.

^a^ p < 0.05.

Note: Models considered autocorrelation and used robust standard errors to account for repeated measures.

No statistically significant level changes were observed in any groups by education level.

## DISCUSSION

The reduction in smoking prevalence from 2006 to 2018 in Brazil is undisputed. Such reduction may be explained by the various tobacco control measures adopted in the country, including forbidding tobacco sale to minors, adding warning labels on cigarette packs, banning tobacco advertising, promotion, and sponsorship, increasing taxation of tobacco products, establishing smoke-free environments, and other measures recommended by the Framework Convention on Tobacco Control (FCTC)^21^.

By analyzing VIGITEL data, we verified an important change in smoking prevalence trend associated with the economic crisis. After the economic crisis onset, in 2015, smoking prevalence immediate increases among people aged 65 years or older when compared with what would be expected if the previous trend had continued. Likewise, the decrease in smoking prevalence decelerates from 2015 to 2018 among men, people aged 18–44 years, and among people with 9 and more years of education, indicating an impact of the economic crisis on smoking behavior.

Our results corroborate those reported by Malta et al.^22^ who found the decreased smoking prevalence in Brazil to decelerate in 2019, urging for a need to monitor fiscal austerity measures implementation, cuts in public spending on social welfare, and the Brazilian government’s weakened regulatory capacity. Despite reporting seminal findings, the authors did not assess whether these changes were significant or related to the economic crisis.

Other studies approach changes in smoking patterns during economic crises. However, to the best of our knowledge, this is the first study to do so in a middle-income country in this level of detail, adding information to the literature on this topic. Other studies reported increased smoking prevalence related to economic downturns, but few analyzed different effects within population groups. In Italy, Mattei et al.^14^ reported increased smoking prevalence associated with the 2008 economic crisis. In the U.S., Gallus et al.^12^ concluded the economic crisis increased the number of adult smokers by 0.6 million. In the U.K., Uphoff et al.^23^ estimated that the 2008-2010 recession was associated with continued smoking during pregnancy. In general, these studies argue that financial pressure increased smoking prevalence due to the supposed stress-reducing effect of smoking.

However, other studies found economic crises to positively influence smoking prevalence. According to Ruhm^24^, every one-point decrease in the employment rate is estimated to reduce smoking prevalence by 0.13 percentage points in the U.S. Jofre-Bonet et al.^25^ interpreted data from 2001–2013 Health Survey for England (HSE) and concluded that the 2008-2010 recession was associated with a decrease in the number of smoked cigarettes. Rathmann et al.^26^ conducted a study with 24 European countries and found higher youth unemployment rates to decrease the likelihood of smoking among adolescents in lower socioeconomic positions. Two studies conducted in Iceland by Ásgeirsdóttir et al.^27,28^ found Icelanders to smoke less during the economic crisis (2007–2009) despite the increased anxiety or poor mental health. These studies postulate that insecure economic circumstances make cigarettes less affordable, consequently reducing smoking prevalence.

The decelerated decline among men under 45 years old and with over 8 years of education may be interpreted as a manifestation of the tension-reduction hypothesis. Yet, we also recognize that stress is not a sufficient reason to start or resume smoking.

Cigarettes prices, along with disposable income, may also justify the differences we encountered between gender subgroups. Paes^29^ conducted a study with Brazilian population and concluded that family income and cigarettes prices influenced women’s decision to quit smoking, but not men.

The immediate increase in smoking prevalence among the oldest group may likewise be explained by the tension-reduction hypothesis. When studying North Americans aged 65 years and older, Shaw at al.^9^ estimated that a one-point increase on a 4-point financial pressure scale was associated with a 12% increase in the likelihood of smoking. The acceleration in the prevalence decrease observed after the immediate increase may reflect changes in individuals’ economic circumstances.

Besides the direct impact of the economic crisis on the population smoking behaviour due to tension reduction or cigarettes affordability, it may also have indirectly influenced such behavior by the consequent weakening of tobacco control initiatives owing to budget cuts or even by enabling a greater influence of the tobacco industry over the government. In Brazil, tobacco prices have not increased since 2017. Moreover, public health surveillance has been diminished in the country, rising the sale of products prohibited by the current legislation, such as single-cigarettes, contraband cigarettes, those lacking health warnings, and e-cigarettes^30^.

Given that these factors may result from political choice rather than from the economic crisis, we cannot dismiss them as an alternative explanation for the decelerated decrease in smoking prevalence from 2015 to 2018.

This study provides new evidence on the association between economic crises and smoking behavior in a middle-income country that has signed the Framework Convention on Tobacco Control and implemented its recommendations.

The 2015–2018 period, characterized by reduced GDP and increased unemployment, experienced an immediate increase in smoking prevalence among people aged 65 years or older, as well as a decrease in the rate of decline for men, people aged under 45 year, and those with more years of education. The differences found among these population groups may be explained by a combination of the tension-reduction hypothesis, the effects of reduced cigarettes affordability, and the weakening of tobacco control measures.

Given that economic crises have different impacts on the smoking habits of different population groups, tobacco control policies should be adapted according to these differences. Control strategies aiming for men, under 45 year old, and with over 8 years of education, should focus on promoting stress-reduction behaviors other than smoking, such as physical exercises. As for women, older people, and those of lower education level, price control measures seem to be more effective.

Our results not only support measures to strength tobacco control and reduce cigarettes affordability, but also call for socially-inclusive economic policies for stress reduction that will exert long-term effects on health-related behaviors rather than solely promote smoking cessation.
